# The psychometric properties of the Bergen–Yale sex addiction scale for the Iranian population

**DOI:** 10.1186/s12888-021-03135-z

**Published:** 2021-03-04

**Authors:** Samaneh Youseflu, Shane W. Kraus, Majid Yousefi Afrashteh, Soudabeh Niroomand

**Affiliations:** 1 Department of Midwifery, School of Nursing & Midwifery, Zanjan University of Medical Sciences, Zanjan, Iran; 2 University of Nevada, Las Vegas, Nevada USA; 3 Department of psychology, University of Zanjan, Zanjan, Iran

**Keywords:** Sex addiction, Bergen–Yale sex addiction scale, Validity and reliability

## Abstract

**Background:**

The assessment of sex addiction among different populations requires a valid and reliable tool. Since the Bergen–Yale Sex Addiction Scale (BYSAS) was not available in Iran, this study aimed to evaluate the validity and reliability of the Persian version of the BYSAS.

**Methods:**

After translation/back-translation procedure, a total of 756 Iranian men and women completed the BYSAS. The structural validity of this tool was evaluated by exploratory and confirmatory factor analyses. An expert panel review also examined content validity of the items. Psychometric properties of the scale including validity, reliability (internal consistency [Cronbach’s alpha]) and test-retest) and factor structure were assessed.

**Results:**

Content Validity Index (CVI) and Content Validity Ratio (CVR) scores for the BYSAS were 0.75 and 0.62, respectively. In the measure’s structural validity, the results of exploratory and confirmatory factor analysis supported the One-factor structure. Data analysis demonstrated satisfactory internal consistency (Cronbach’s alpha ranging from 0.88 to 0.89).

**Discussion:**

Study findings suggest that the BYSAS is a valid and reliable instrument for assessing sex addiction among Persian speaking adults. Replication of research findings is needed to expand the BYSAS for clinical and non-clinical Iranian populations.

## Background

Sexual addiction is usually defined as any sexually-related, compulsive behavior which interferes with normal living and causes severe stress on family, friends, and one’s work environment [1, 2].

Sex addiction symptoms consist of looking for new sexual partners, having compulsive masturbation and sexual intercourse, frequent pornography consumption, repeated unsuccessful efforts to stop excessive sexual behaviors, having risky sexual experiences, obsessive thoughts of sex, feeling guilt or shame about their sexual behaviors, and a strong desire for anonymous sex [1, 3–5]. Sex addiction is associated with increased risk-taking behaviors such as drug use, alcohol consumption and having multiple sexual partners, anxiety, depression, impulsivity, loneliness, low self-confidence, and insecure attachment styles [1, 3, 6–8].

Although the latest edition of the Diagnostic and Statistical Manual of Mental Disorders (DSM-5) did not accept sex addiction as a mental disorder, excessive sexual behavior conceptualized as a “Sexual Disorder Not Otherwise Specified” is in the DSM-III.

In the general population, 15–61% of people assessed by various questionnaires [9–11], were estimated as sex-addicted [8]. The prevalence of this disorder among Iranian population is unknown.

Regarding the traditional and religious culture of Iranian society, some sexual activity (erotic behavior, masturbation) and cross-gender interactions such as premarital dating and friendship of girls and boys are legally banned and culturally unacceptable [12]. Hence, these issues make many problems for these patients. On the other hand, due to the lack of a valid and reliable instrument to diagnose this disease, many research about them are limited. Therefore, we need a valid and reliable tool in line with Iranian contexts.

The Bergen–Yale Sex Addiction Scale (BYSAS) is a short self-report instrument that specifically measures sexual addiction and it is available in English and Norwegian languages. Andreassen and colleagues developed and validated this scale in 2018 [9]. To expand the utility of the BYSAS for diverse populations, the aim of the current study was to translate and investigate the psychometric properties of the BYSAS in a sample of Iranian adults.

## Methods

### Participants

This cross-sectional study was conducted in Zanjan University of Medical Sciences, Zanjan, Iran, from February to November 2019. The ethics committee approved the study (1397.328IR.ZUMS.REC.). The inclusion criteria for participant selection included: 1) age between 18 and 60 years; 2) necessary communication skills; 3) the absence of intellectual disability; and 4) not using drugs, and medications affecting sexual functioning. Participants were 756 Iranian men and women, aged up to 60 years who were selected based on convenience sampling method. Sampling was performed at 8 to 11 am and 5 to 9 pm in summer and winter to avoid the influence of seasonal and temporal conditions on the sexual behavior. Before completing the questionnaire, verbal information was provided to inform participants about the purpose of the study and adherence to ethical principles and informed consent form was obtained. According to application of exploratory and confirmatory analyses, the study sample was divided into 376 and 380 individuals. Sample information is shown in Table 1.

**Table 1:** Participants’ characteristics (*N* = 756)

Variables	First sample	Second sample
Age g	34.31(8.22)	33.95(8.67)
Gender m		
Male	128(34%)	144(38%)
Female	248(66%)	236(62%)
Marital status		
Single	90(23.9%) 271	100(26%) 271
Married	273(72.9%)	266(70%)
Others	12(3.2%)	14(4%)
Job		
Employment	162(43.1)	164(43.2)
Worker	12(3.2)	10(2.6%)
Self-employment	56(14.9)	58(15%)
Jobless	27(7.2)	28(7.3)
House wife	119(31.6)	120(31.5)
Education		
University	240(63.8)	252(66.3%)
Non university	136(36.2)	128(44.7%)

### Measures

#### The Bergen–Yale sex addiction scale

The Bergen–Yale Sex Addiction Scale (BYSAS) consists of 6 statements. The BYSAS is scored by adding the score of each single item (0 = very rarely, 1 = rarely, 2 = sometimes, 3 = often, and 4 = very often). The BYSAS yields a composite/continuous score ranging from 0 to 24. Hence, the BYSAS can be used as a continuous score of sex addiction. To classify a respondent as a “sex addict”, at least 4 symptoms have to be present at a specific level/magnitude [defined as scoring at least 3 (often) or 4 (very often)]. Hence, the BYSAS can also provide a dichotomized score/categorization. Indeed, a specific number of criteria (often more than half) had to be approved (here “often” or “very often”) to be classed as having sex addiction. Also, participants that answered “never” to all the six items were classified as having “no sex addiction”. A summed score between 1 and 6 (two of the six items) was considered as “low sex addiction risk”. Those with a composite score of 7 or above but did not fulfill the criteria for sex addiction were defined as having “moderate sex addiction risk”. This label seems suitable as this equals a mean score above one on all six items [9].

#### Translation

The ‘forward-backward’ procedure was applied to translate the questionnaire from English into Persian (Iranian language). Two health professionals translated the questionnaires into Persian and these were backward translated into English by a psychologist and a professional translator. Then, a provisional version of the Iranian questionnaire was provided. In general, there were no difficulties in translating items.

### Statistical analysis

The data were analyzed using SPSS version 16 for windows (SPSS, Inc., Chicago, IL, USA) and Lisrel 8.8 software was used for confirmatory factor analysis. The weighted least squares method with data from the correlation coefficient and asymptotic covariance matrix was used for data analysis. The indices used for the confirmatory model are as follows: X^2^ Exponential Ratio (X^2^), X^2^ Liberty Ratio (X^2^ / df), Goodness of fit index (GFI), Adjusted Goodness of fit Index (AGFI), Root Mean Square Error of Estimation (RMSEA), CFI and NFI and Tucker-Lewis Index (TLI). In addition, Cronbach’s alpha was used to assess the internal consistency coefficient of the scale.

Face validity, content validity, exploratory factor analysis (in the first sample), and confirmatory factor analysis (in the second sample) were used to assess the validity and Cronbach’s alpha coefficient and test-retest reliability.

#### Validity

##### Face validity

Face validity is the objective judgment of the instrument and responds from the point of view of the target group that is the designed tool seemingly relevant to the purpose of the study? Do people who want to respond to the tool, agree with the tool’s wording and expressions? Is the perception of non-specialists (the target group) the same as the researcher intended? Are the components and totality of the instrument acceptable to respondents? In this study, qualitative and quantitative methods were used to determine face validity of the instrument. In the qualitative method and at the beginning of the face validity determination process, a team of psychologists and psychometrists with relevant research backgrounds were asked to evaluate the tool questions to determine the appropriateness of the words and sentences for the target group. In some cases, items were changed to make them easier and more understandable.

To get the target group’s comments, an interview was also conducted with a sample of participants to identify difficulty in understanding the wording and phrasing of items, the appropriateness and relevance of the items, the likelihood of ambiguity and inaccurate interpretations of the phrases, or the inaccuracy of word meanings and their comments were made as minor changes to the questionnaire. The face validity of the measures was quantitatively by the item impact method. For this purpose, a 5-point Likert scale was considered for each item of the measure: extremely important (5 points), important (4 points), moderately important (3 points), slightly important (2 points) and not at all important (1 points). Then, the questionnaire was administered to 20 individuals of the group to determine face validity and after completing the questionnaires, face validity was calculated using an item impact formula. To accept the face validity of each item, its effect score should not be less than 1.5 and only face validity questions are acceptable which is a score above 1.5.

##### Content validity

Content validity answers questions such as: does the designed tool include all the important aspects of the concept of measurement? Does the tool item measure what it should? In this study, both qualitative and quantitative methods were used to determine content validity. A qualitative method was consulted with clinical psychology and psychometrics specialists about quality and adequacy of items. After collecting the expert evaluation, the required changes to the tool were considered and applied in consultation with the members of the research team. Content validity was calculated quantitatively based on the experts’ opinions and by calculating both content validity ratio (CVR) and content validity index (CVI).

The content validity ratio was used to ensure that the most important and correct content (item necessity) was selected and the content validity index was used to ensure that the tool items were best designed to measure content. The content validity ratio quantitatively was used by 10 experts in clinical psychology and psychometrics who were all faculty members of Iranian universities in order to respond to each of the tool items or metrics used in the three item ranges (including essential, useful, but not necessary, and not necessary).

The referee’s opinion was calculated as follows:

$$ CVR=\frac{\left( ne-\frac{N}{2}\right)}{\frac{N}{2}} $$

The formula for the content validity ratio is the number of evaluators who consider the item necessary or useful and N is the total number of evaluators or reviewers who have reviewed the item.

The CVI index was calculated after determining the CVR. To calculate this index, evaluators commented on each item of the instrument used (the Bergen–Yale Sex Addiction Scale), based on the four criteria of relevance or propriety, simplicity and fluency and clarity or transparency, based on a 4-point Likert scale. For example, the criterion was used for the relevance of options (not relevant = 1, relatively relevant = 2, and relevant = 3 and fully relevant = 4). Then, the content validity index was calculated using the CVI formula.

$$ CVI=\frac{ne_{3,4}}{N} $$

ne3, 4: The number of ratings given the score of 3 and 4.

N: Total number of evaluators.

The minimum score required for acceptance of content validity ratio (CVR) according to the Laosche method for 10 expert members was 0.80 and the minimum content validity index (CVI) was 0.75.

#### Structural validity

A confirmatory factor analysis was conducted to confirm the factor structure reported in the first sample. If the structure obtained from the first sample to the second sample is confirmed, a certificate is provided for its validity. Lisrel8.8 software was used for confirmatory factor analysis. Weighted Least Squares estimation method was used in data analysis with data from polychoric correlation and asymptotic covariance matrix. The Weighted Least Squares method was preferred because the query options were four-class and Polyureic correlation was calculated instead of Pearson correlation [13]. There are two types of evaluations to consider in confirmatory models. Partial evaluation and overall fit of the model. The Partial evaluation relates to paths drawn from current agents to the markers (in measurement model). The overall fit of the measurement models was judged using several goodness of fit indices (which measure the amount of data support for the conceptual model). The indices used are: Chi-square exponential ratio (X2), Chi-square to degrees of freedom (X2 / df), goodness of fit (GFI), modified goodness of fit (AGFI), root mean square error of estimation (RMSEA), CFI and NFI and the Tucker-Lewis Index (TLI).

## Results

### Participants

The primary research aim was to investigate the psychometric properties of the BYSAS. The characteristics of the participants are shown in Table 1. The mean score of participant’s age in the first, and second sample were 34.31 + 8.22, and 33.95 + 8.67, respectively. The majority of participant (about 70%) were married, and more than 60% of them had university education. More than 40% of participants at the first, and second sample were employment.

### Face and content validity

The results obtained from Table 2 shows that all items reported have acceptable face validity with scores more than 1.5. Table 3 shows the content validity of questionnaire. The minimum score required for acceptance of content validity ratio (CVR) according to the Laosche method for 10 expert members was 0.80 and the minimum content validity index (CVI) was 0.75. According to the results, all items on the scale had good validity.

**Table 2:** Result of the face validity

Item no.	Effect size	n_e_/N	Mean	not at all important (1 points)	slightly important (2 points)	moderately important (3 points)	important (4 points)	Extremely important (5 points)
**1.** Spent a lot of time thinking about sex/masturbation or planned sex?	2.96	0.75	3.95	0	1	3	7	8
**2.** Felt an urge to masturbate/have sex more and more?	3.61	0.85	4.25	0	1	2	8	9
**3.** Used sex/masturbation in order to forget about/escape from personal problems?	3.24	0.80	4.05	1	1	2	8	8
**4.** Tried to cut down on sex/masturbation without success?	3.28	0.80	4.10	3	2	2	8	8
**5.** Become restless or troubled if you have been prohibited from sex/masturbation?	3.28	0.80	4.10	0	2	2	8	8
**6.** Had so much sex that it has had a negative impact on your private relationships, economy, health or job, studies?	3.82	0.90	4.25	0	2	0	9	9

**Table 3:** Result of the content validity

Item no.	Ne	CVI	CVR
1	10	0.916	1
2	10	0.966	1
3	9	0.933	0.8
4	10	0.941	1
5	10	0.933	1
6	9	0.908	0.8

### Exploratory factor analysis

The results of exploratory factor analysis are reported in Table 4. To determine the number of factors, the Kaiser criterion was used which retains factors with eigenvalues greater than 1. Only one factor has this criterion. Principal component was used for data analysis and varimax rotation was used for extraction of factors. Data from 376 participants (34% male and 66% female) was used for this phase. Only 8 questionnaires were incomplete. Four questionnaires were excluded from the study due to participant’s withdrawal from the study in order to adhere to ethical principles and avoid selective bias. Data on 364 questionnaires were analyzed. The eigenvalue was obtained for 3.74, which explains the variance of 62.31. No questions were deleted and the load factor of the remaining questions is between 0.76 and 0.84.

**Table 4:** One-factor exploratory factor analysis results for the BYSAS

Item no.	Subscription rate	factor loadings
1	0.71	0.83
2	0.64	0.80
3	0.63	0.77
4	0.69	0.84
5	0.59	0.80
6	0.52	0.76

% of Variance: 62.31, Eigenvalue: 3.74

As Table 4 shows, all factor loadings on items were higher than 0.7, indicating that the questions are significantly related to their underlying factor. As mentioned above, with respect to the sample size, the factor loadings above 0.40 are significant. The bottom column of the table above shows the subscription rate for each of the questions. This value represents the amount of variance explained by each factor extracted. For example, 69% of the variance of the first question is explained by the underlying factor. Below the table is reported the specific amount and variance explained by the underlying factor. The eigenvalue represents some of the total variance of the variables by which the agent is explained. The total amount of variance explained in the model is 62.31%, reflecting a unidimensional factor measuring sex addiction.

**Fig. 1:**
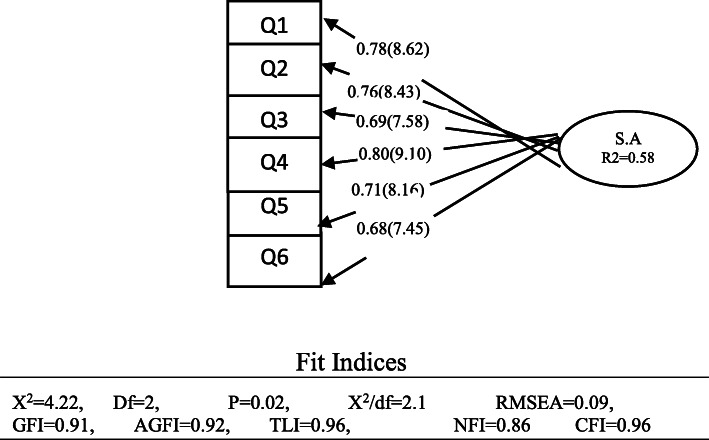
Standard Rate and T-value for confirmatory factor analysis of BYSAS

Figure 1 shows the results of the confirmatory factor analysis and the fit indices. These indicators provide the information to evaluate the overall fit of the model. The fit indices support the proposed one-factor model. According to the information in this table, out of the eight indices reviewed, six indices are in favorable condition and only NFI and RMSEA are in relatively favorable condition. Based on the results, it can be said that the overall fit of the measurement model is in the optimum condition.

#### Reliability

Cronbach’s alpha coefficient of internal consistency for the instrument was 0.88 (95% confidence intervals: 0.83–0.83), suggesting the scale has good internal consistency. The mean inter-item correlation was 0.54. In addition, the intra-class correlation coefficient was obtained between the two test runs with 2 weeks intervals for 23 persons equal to 0.89, which is an acceptable value (*p*-value = 0.009). Therefore, it shows that the reliability of sex addiction test is valid.

## Discussion

To our knowledge, this study is the first assessment of a sex addiction measure ensuing from translating, conducting reliability and testing validity of the BYSAS in a non-clinical sample in an Iranian population. Generally, participants found the BYSAS to be fast and clear to complete, and reflective of their experiences. The scale also demonstrated excellent psychometric properties (high internal consistency, test-retest reliability, and evidence of construct validity).

As noted by others, translating an existing tool into another language is not a simple translation of words and requires extensive research to produce a cultural version comparable to the tool plus psychometric testing to ensure equivalence [14, 15]. In this study, cultural and conceptual equality were obtained; furthermore, we used guidelines to examine the psychometric properties of the instrument. The Persian version of the BYSAS was culturally functional and transferred the main purpose of the original English BYSAS.

In this study, we found acceptable validity (face and content) and reliability. In terms of face validity, experts and other interviewees (males and females) read the BYSAS items using similar interpretations. Respondents said that the questionnaire covers all aspects of sexual addiction. We also found that the impact score of all items was above 1.5 that indicates the proper face validity of the translated tool (especially Persian). However, the instruments used in previous studies were inappropriate with regard to defining sex addiction [16]. The shortcomings of the previous instruments were offset by the construction of this questionnaire by Anderson et al., and its validity was confirmed by our study. Specifically, we found that a single factor accounted for 62.31% of the total variance. Also, the instrument yielded high content validity that was consistent with previous studies, despite different sample sizes, race or cultures, and different translations (such as the study of Paz et al., (Hebrew), and Anderson et al., (original English version)) [9, 17]. However, in comparision with other countries, Iranians might have the same experience of sexual addiction.

According to the psychometric properties of this study, the BYSAS was highly test-retest reliable (a 2-week period) for screening of sexual addiction and Cronbach’s alpha was for internal consistency coefficient of 0.88 and for the intra-class correlation coefficient of 0.89 that was higher than the values ​​obtained in previous reliable studies [9, 17]. In addition, the positive and significant correlations found in the Christon et al. study indicated the high reliability of the BYSAS. Also, Paz et al. showed high test-retest reliability of the instrument, but covariances emerged in the model of his study could ensue from the smaller sample size or interpretation features in Hebrew.

Moreover, the results concerning the one-factor structure analysis model with six subscales are consistent with the previous studies funding from Norway [9] and Italy [18] that investigated the psychometric properties of the instrument. Also, our study indicated that the favorable relationship of all markers of the model with their substrates and strong association between its subscales, and appears suitable for the evaluating of the partial and general indices and specifically known for sexual addiction. In studies, there are slight differences due to different age groups (variety in cognitive abilities between adolescents and adults, (different countries (different habits or accessibility to the social media) and cultural variation.

The current study presents a number of limitations. First, the study is limited by the common limitations in the psychological literature, including using voluntary partisipation (e.g. the self-selected samples) and self-report data. Second, despite the adequacy of the sample size, it was not nationally representative. Third, for the validity assessment of this scale, we used face, content, and construct (only confirmatory factor analysis) validity. We also did not assess other types of construct validity such as concurrent, discriminant, predictive, convergent, and criterion-related validity. Forth, the use of convenience sampling method is other limitation of this study. Future research is needed to examine the clinical utility of the BYSAS among clinical populations in Iran.

## Conclusion

Our findings illustrate that the Persian version of the BYSAS is appropriate for assessing sex addiction in Iranian population ensuing from the initial reliability and validity in a general sample.

## Data Availability

The data sets used and analyzed during the current study are available from the corresponding author on reasonable request.

## Abbreviations

BYSAS: Bergen Yale Sex Addiction Scale
RMSEA: Root mean square error of approximation
AGFI: Adjusted Goodness of Fit Index
CFI: Confirmatory Factor Analytic
DF: Degree of Freedom
TLI: Tucker-Lewis Index
