# Correction to: The psychometric properties of the Bergen–Yale sex addiction scale for the Iranian population

**DOI:** 10.1186/s12888-021-03172-8

**Published:** 2021-04-29

**Authors:** Samaneh Youseflu, Shane W. Kraus, Fatemeh Razavinia, Majid Yousefi Afrashteh, Soudabeh Niroomand

**Affiliations:** 1 Department of Midwifery, School of Nursing & Midwifery, Zanjan University of Medical Sciences, Zanjan, Iran; 2 University of Nevada, Las Vegas, Nevada USA; 3 Department of Reproductive Health and Midwifery, Faculty of Medical Sciences, Tarbiat Modares University, Tehran, Iran; 4 Department of psychology, University of Zanjan, Zanjan, Iran

**Correction to: BMC Psychiatry 21, 126 (2021)**

**http://dx.doi.org/10.1186/s12888-021-03135-z**

Following the publication of the original article [1], the authors identified a missing co-author in the published article. The name of the author is given below.

Fatemeh Razavinia

The author group has been updated above and the original article [1] has been corrected.
